# Special attention to nurses’ protection during the COVID-19 epidemic

**DOI:** 10.1186/s13054-020-2841-7

**Published:** 2020-03-27

**Authors:** Lishan Huang, Guanwen Lin, Li Tang, Lingna Yu, Zhilai Zhou

**Affiliations:** 1 The Spine Department, Orthopaedic Center, Guangdong Second Provincial General Hospital, Guang Zhou, Guangdong Province China; 2 Infection Control Department, Guangdong Second Provincial General Hospital, Guang Zhou, Guangdong Province China; 3 Nursing Department, Guangdong Second Provincial General Hospital, Guang Zhou, Guangdong Province China

## Abstract

As of March 8, 2020, the novel coronavirus disease 2019 (COVID-19) had caused 80,815 human infections and 3073 deaths in China, including more than 3000 infections among medical staff. Guangdong Second Provincial General Hospital (Guangzhou, Guangdong Province, China), a provincial emergency hospital, has treated more than 35 confirmed cases of COVID-19 and more than 260 suspected cases. Most of nurses’ work involves direct contact with patients. As nurses have high vulnerability to COVID-19, it is necessary to establish hospital-specific protocols to reduce the risk of nurses’ infection in interactions with COVID-19 patients. Our hospital has maintained a “zero nurse infection” rate while battling SARS in 2003 and during the present COVID-19 epidemic. The following are the key measures implemented in our hospital.

## Provide intense education and training for nurses

We provide adequate education to nurses, and training content includes the use of personal protective equipment (PPE), hand hygiene, ward disinfection, medical waste management, and sterilization of patient-care devices and management of occupational exposure. The PPE sets include (listed in the order they are to be put on) a disposable work hat, an N95 respirator, inner gloves, a protective eye mask, protective clothing, disposable waterproof shoe covers, disposable isolation gowns, outer gloves, and a face shield. Due to the complicated procedure of putting on and removing the PPE, we recorded a teaching video and sent it to a WeChat group where all nurses could review the operation details any time.

## Establish a scientific, reasonable shift schedule

With the rapid increase in patients, which will cause severe nurse shortages, it is extremely important to establish a scientific, reasonable nursing shift schedule. We have tried 3 shift schedules: (1) 4 h of work in the morning and 4 h of work in the afternoon with an 8-h interval; (2) 6 h of continuous work; and (3) 6 h of continuous work, with the next nursing shift overlapping by 1 h at the end of shift. After a 1-week trial of the different shifts, a questionnaire survey was conducted among 78 nurses to investigate their preferred shift schedule and their reasons. The results showed that 74% of nurses preferred the third schedule for the following reasons: (1) putting on and taking off PPE twice a day increased medical resource consumption; (2) frequently moving between contaminated and clean areas increased the risk of infection; (3) frequently going through the complicated procedures of putting on and taking off PPE increased their mental burden; (4) working for 6 h continuously pushed their physiological limits, as they could not go to the bathroom when wearing PPE in the isolation area, and they often felt groggy or tired at the end of work; and (5) having 1 h of overlap between shifts provided flexibility and facilitated the handover, which reduced nurses’ mental stress and the possibility of adverse events. In addition, the 1-h overlap allows two nurses to cooperate in finishing tasks that are difficult for a single person to complete, such as administering injections to and drawing blood from children, changing sheets, and performing terminal room disinfection.

## Take full advantage of the infection control system

**Fig. 1:**
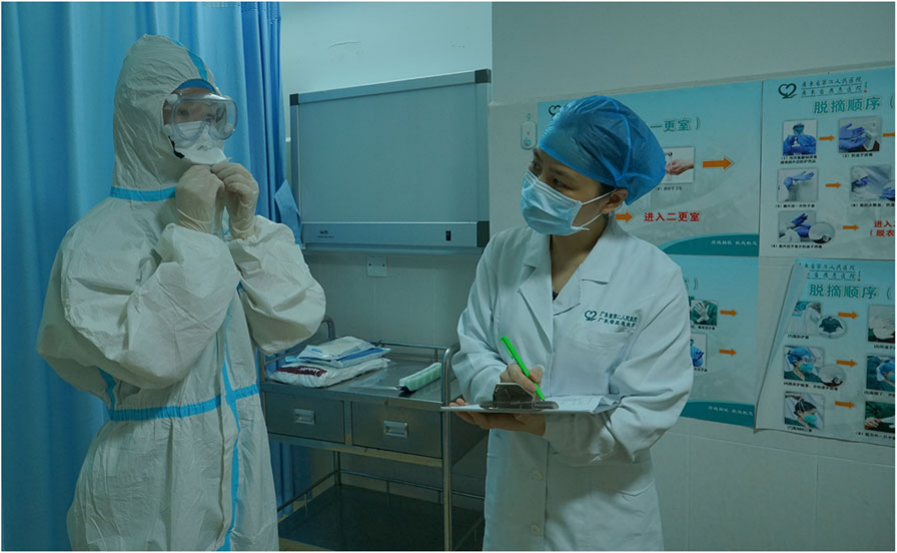
An observer providing instructions to a nurse wearing PPE

Despite intense training, it is not uncommon for nurses to not be fully aware of their exposure while caring for patients, especially when they feel stressed or exhausted. Our hospital has established an infection control system called the observing system that provides real-time monitoring and aids in instant correction [1]. The observers usually monitor medical staff in real time on computer monitors in a separate area; sometimes, they also monitor nurses face-to-face if necessary, such as while nurses take off and put on PPE (Fig. 1). In our experience, nurses are more prone to make mistakes when entering or leaving the isolation area. For example, before the observing system was introduced, a nurse in our department put on the PPE in the wrong order; she put on the isolation gowns before the goggles (which should be put on before the isolation gowns), which led to her exposure when she took off the PPE. To eliminate the possibility of infection, she had to be isolated for 14 days.

## Provide psychological counseling

The risk of COVID-19 infection may cause significant psychosocial stress for medical staff. Unfortunately, several young medical staff members infected with COVID-19 whose cases appeared to be mild at the early stage of the disease recently sharply deteriorated and died, further increasing the fear of the virus. To relieve the mental stress of nurses, the head nurse has a 30-min meeting with nurses who will work in the isolation area on the next day to make them aware of the adequate equipment and resources in our hospital, the observers who will send instant aid if necessary, etc. Furthermore, nurses are protected and evaluated the first moment they feel any discomfort; nurses with symptoms of anxiety or insomnia are encouraged to seek help from psychotherapists on our team on duty 24 h a day who will evaluate them and help them deal with potential stress and depression.

## Avoid unnecessary contact

Avoiding unnecessary contact is critical for minimizing cross-transmission. Our department is equipped with well-established hospital information systems, personal digital assistant (PDA) systems, and a local intranet. All wards are entirely monitored by cameras so we can achieve the following:

1. All medical documents including physicians’ order sheets, medical records, consent information, examination results, and nursing materials are paperless.
2. Nurses and doctors can monitor the situation in each room in real time, and depending on the situation, doctors and nurses can give remote assistance to avoid unnecessary contact.

## Conclusion

In conclusion, COVID-19 is a highly contagious disease, hospital-related transmission of the virus is still a very large threat to health-care workers, and nurses are at the front lines of care and are thus more susceptible to infection. We believe that a flexible, adjustable policy and protocols play a vital role in reducing nosocomial infection.

## Data Availability

Not applicable.

## References

[1] Chen X, Tian J, Li G, Li G. Initiation of a new infection control system for the COVID-19 outbreak. Lancet Infect Dis. 2020. PMID: 32085850. 10.1016/S1473-3099(20)30110-9. [Epub ahead of print]. 10.1016/S1473-3099(20)30110-9 PMC7129690 32085850

